# Connecticut

**Published:** 1896-10

**Authors:** 


*Connecticut.* Connecticut laws demand that all cattle shipped into the State shall be tested with tuberculin and have a certificate from a reputable veterinarian that they are free from tuberculosis. Thirty-six cattle recently purchased in Sullivan County, New York, underwent the test recently before shipment, by which the raisers of cattle in this county hope again to regain the market in Connecticut for the sale of their animals.

A herd of eighteen cattle at West Hartford were recently tested, nine of the number proving tuberculous.

A load of cattle was recently shipped from Maine to Connecticut, and there tested with tuberculin; one responded to the test, and was killed. This herd was the source of an action at law, because it was brought into the State without a permit, though the cattle were immediately quarantined, and the commission notified, when they reached their destination in the "Nutmeg State."

